# In vivo adenine base editing reverts C282Y and improves iron metabolism in hemochromatosis mice

**DOI:** 10.1038/s41467-022-32906-9

**Published:** 2022-09-05

**Authors:** Alice Rovai, BoMee Chung, Qingluan Hu, Sebastian Hook, Qinggong Yuan, Tibor Kempf, Florian Schmidt, Dirk Grimm, Steven R. Talbot, Lars Steinbrück, Jasper Götting, Jens Bohne, Simon A. Krooss, Michael Ott

**Affiliations:** 1 Department of Gastroenterology, Hepatology and Endocrinology, Hannover Medical School, Hannover, Germany; 2 Twincore Centre for Experimental and Clinical Infection Research, Hannover, Germany; 3 Department of Cardiology and Angiology, Hannover Medical School, Hannover, Germany; 4 Department of Biosystems Science and Engineering, ETH Zürich, Basel, Switzerland; 5 Department of Infectious Diseases/Virology, Medical Faculty, University of Heidelberg, Cluster of Excellence CellNetworks, BioQuant BQ0030, Im Neuenheimer Feld 267, 69120 Heidelberg, Germany; 6 BioQuant, Center for Integrative Infectious Diseases Research (CIID), University of Heidelberg, Heidelberg, Germany; 7 German Center for Infection Research (DZIF) and German Center for Cardiovascular Research (DZHK), partner site Heidelberg, Heidelberg, Germany; 8 Institute for Laboratory Animal Science and Central Animal Facility, Hannover Medical School, Hannover, Germany; 9 Institute of Virology, Hannover Medical School, Hannover, Germany

**Keywords:** CRISPR-Cas9 genome editing, Molecular medicine, Metabolic disorders, Hepatology

## Abstract

Hemochromatosis is one of the most common inherited metabolic diseases among white populations and predominantly originates from a homozygous C282Y mutation in the *HFE* gene. The G > A transition at position c.845 of the gene causes misfolding of the HFE protein, ultimately resulting in its absence at the cell membrane. Consequently, the lack of interaction with the transferrin receptors 1 and 2 leads to systemic iron overload. We screened potential gRNAs in a highly precise cell culture assay and applied an AAV8 split-vector expressing the adenine base editor ABE7.10 and our candidate gRNA in 129-Hfe^tm.1.1Nca^ mice. Here we show that a single injection of our therapeutic vector leads to a gene correction rate of >10% and improved iron metabolism in the liver. Our study presents a proof-of-concept for a targeted gene correction therapy for one of the most frequent hereditary diseases affecting humans.

## Abstract (web summary)

Hemochromatosis is a metabolic disorder caused by mutations in the HFE gene. Here, the authors show that a single administration of AAV8 vectors expressing an Adenine Base Editor facilitates efficient in vivo gene correction in hepatocytes and leads to improvement of iron-specific parameters in the liver and the blood in mouse models of the disease.

## Introduction

Hereditary hemochromatosis is caused by mutations in genes involved in the regulation of systemic iron homeostasis. Dysregulation of iron homeostasis leads to the absorption and storage of more iron than required, causing tissue damage and disease. Five genes have been associated with various subtypes of the disorder. The most common gene involved in hereditary hemochromatosis is *HFE* (OMIM 235200). Homozygosity for a single missense substitution (p.Cys282Tyr) is responsible for most cases among European populations. The G > A transition at position c.845 of the gene causes the disruption of an intrachain disulfide bond, which leads to misfolding of the HFE protein. As a result, the mutated protein cannot associate with beta-2 microglobulin and does not reach the cell membrane, where it interacts with the transferrin receptors 1 and 2 (TFR1,2)^1^. This consequently leads to continuous iron uptake in the intestine and accumulation of iron in various organs such as the liver, pancreas, and heart. Affected patients may exhibit liver cirrhosis, diabetes mellitus as well as cardiomyopathy. The current state-of-the-art treatment of hemochromatosis includes life-long phlebotomy and the application of chelating agents such as deferoxamine.

With the rapid emergence of novel CRISPR/Cas-based gene-editing tools, proof-of-concept gene therapy strategies were rapidly developed. Metabolic liver diseases such as ornithine transcarbamylase deficiency, tyrosinemia type 1, and alpha-1-antitrypsin deficiency were successfully treated in vivo in various mouse models^2–5^.

Recently, the adenine base editing system ABE7.10 was developed, facilitating the highly efficient conversion of A•T to G•C^6^. This editing tool represents a powerful instrument for future therapy of genetic disorders, since according to the *ClinVar* database (https://www.clinicalgenome.org/data-sharing/clinvar/), ~48% of pathogenic human single nucleotide polymorphisms (SNPs) can be corrected by A•T > G•C transitions^7^. Furthermore, as the targetable adenine deaminases do not require DNA double-strand breaks and therefore lower the risk of, e.g., chromosomal translocations^8^, this gene-editing approach holds enormous potential for clinical application.

Recombinant adeno-associated virus (rAAV)-based vectors have been frequently used for the delivery of genes since natural and synthetic isolates exhibit a broad spectrum of organ specificities^9^. Recent clinical trials have underlined the therapeutic efficiency and safety of these vector systems for the treatment of diseases such as haemophilia B^10^ or Leber’s congenital amaurosis^11^. However, a major draw-back of rAAV remains the size limitation of the transgene, since the commonly accepted maximum cargo size of AAV amounts to 4.9 kb^12^ or 5 kb^13^, which largely limits the incorporation of genes such as programmable nucleases. A potential solution to the cargo size problem was presented with the development of split-vector systems, which facilitate the fusion of two separate gene fragments into a functional mRNA, e.g., via *trans*-splicing^14,15^. Recently, the ABE7.10 system was delivered via AAV split-vectors to correct a disease-causing mutation in the dystrophin gene of mice^16^.

In this study, we present an in vivo gene editing approach to correct the C282Y (c.845G > A) mutation in the *Hfe* gene in the 129-Hfe^tm.1.1Nca^ mouse model using the adenine base editor ABE7.10 delivered by an AAV8 split-vector. Our results indicate that a single application of the therapeutic vector leads to gene correction rates of ~10% and significant improvement of iron-associated parameters in blood and liver tissue.

## Results

### A GFP-based reporter system enables precise gRNA screenings

**Fig. 1:**
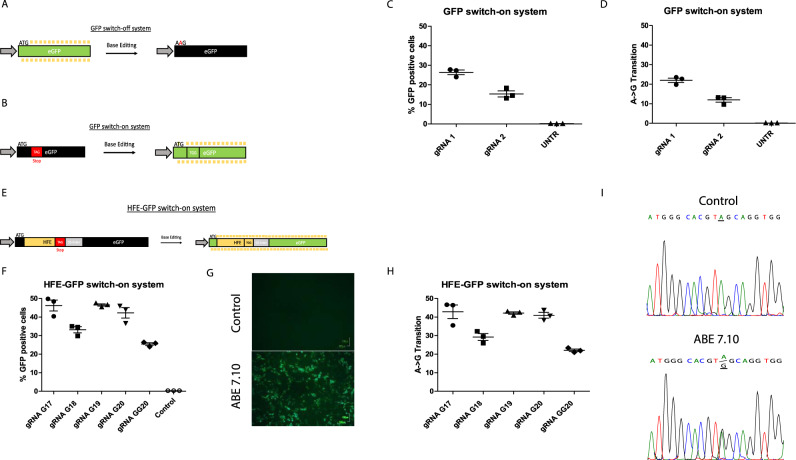
Cell culture-based base editing reporter system. **A** Depiction of GFP switch-off system. The start-ATG of the ORF is targeted by a gRNA directing ABE7.10. Upon editing, ATG will be converted to GTG, leading to the inactivation of GFP expression. **B** Depiction of GFP switch-on system. Twenty nucleotides downstream of the start-ATG, a premature stop codon TAG was inserted leading to a nonfunctional GFP. Base editing will result in the reconstitution of the *gfp* ORF. **C** Flow cytometric determination of GFP-positive HEK-293T cells (GFP switch-on system) upon transfection with gRNAs and ABE7.10 (*n* = 3 biologically independent samples). The bars in the scatter dot plot depict the mean with the SD**. D** Determination of base conversion rate via NGS. The *gfp* locus was PCR-amplified and subjected to NGS (*n* = 3 biologically independent samples). The bars in the scatter dot plot depict the mean with the SD**. E** Depiction of the HFE-GFP switch-on system. Into the *gfp* ORF, the *HFE* target sequence including a premature stop codon (TAG) was inserted. A GS-linker was inserted to protect the fluorophore function. Upon base editing, the premature stop codon is converted into TGG, thereby reconstituting the *gfp* ORF and fluorescence. **F** Flow cytometric analysis of GFP activity in retrovirally transduced HFE-GFP switch-on HEK-293T cells upon transfection of ABE7.10 and gRNAs of various lengths (*n* = 3 biologically independent samples). The bars in the scatter dot plot depict the mean with the SD**. G** Fluorescence microscopy of GFP-positive cells described in **F** after base editing. **H** Determination of C282Y correction rate via NGS. The *HFE* target locus was PCR-amplified and subjected to NGS (*n* = 3 biologically independent samples). The bars in the scatter dot plot depict the mean with the SD**. I** Chromatogram of corrected *HFE* target sequence and untreated cells. The *HFE* target locus was sequenced via Sanger sequencing. The underlined nucleotides indicate successful base editing. Source data are provided as a Source Data file.

To first evaluate the efficacy of our base editing system, we created GFP-based reporter cell lines, which immediately indicate genome editing. We initially generated a HEK-293T cell line stably expressing a *gfp* gene to be targeted by the ABE7.10 system (Fig. 1A). Upon base editing, we expected to detect a decrease in GFP expression via flow cytometry. However, this GFP switch-off system presented highly fluctuating results (Supplementary Fig. 1A). Therefore, we modified our reporter system into a GFP switch-on system, which carries a premature stop codon within the first 20 nucleotides of the *gfp* sequence (Fig. 1B). This stop codon was then successfully edited upon transfection of ABE7.10 DNA and gRNAs of different lengths, based on the finding of previous reports that truncated gRNAs can optimise gene editing efficiency^17^. The reconstitution rate of the *gfp* open reading frame and subsequent GFP expression was up to 26% ± 2.07 as determined via flow cytometry (Fig. 1C). Next Generation Sequencing (NGS) analyses of the edited *gfp* locus were performed to assess the precision of the GFP switch-on system, which revealed results similar to those observed via flow cytometry (Fig. 1D).

Having validated the precision and efficiency of ABE7.10 in our GFP switch-on system, we aimed to conduct gene editing and gRNA screening on the *HFE* locus. Therefore, we positioned the *HFE* target sequence carrying the human c.845G > A mutation within the premature stop codon 10 bp downstream of the start ATG of the *gfp* gene. For this purpose, the sequence TAT, characteristic of hereditary hemochromatosis, has been modified into the stop codon TAG. This modification does not interfere with the activity of ABE7.10 and it would give rise to an inactive *gfp* transcript. To avoid interference of the *HFE* target sequence with the fluorophore activity of GFP, we inserted a glycine-serine linker sequence (Fig. 1E). To validate that the insertion of the *HFE* sequence and the glycine-serine linker still permits GFP function, a variant of the *HFE* target sequence without a stop codon was generated. This control vector presented unrestricted GFP activity as confirmed via fluorescence microscopy (Supplementary Fig. 1b). Next, several gRNAs with different lengths and numbers of leading Gs were designed and transfected into the HFE-GFP reporter cell line with the ABE7.10 plasmid. Editing efficiency was evaluated by measuring GFP expression via flow cytometry (Fig. 1F, G) and by NGS (Fig. 1H). Notably, both read-out methods achieved highly similar values for gene editing among the different gRNAs, which underlines the precision of our HFE-GFP switch-on system. A duplication of the leading G neither led to enhanced editing efficiency nor the truncation of the gRNA to 18 nucleotides. Finally, we observed superior gene editing efficiency for gRNA G17 (46.2% ± 5.07), G19 (46.5% ± 1.05) and G20 (42.27% ± 4.91) (Supplementary Table 2). Based on human and mouse sequence similarity, the results obtained from the HFE-GFP switch-on system can be translated to the murine mutation.

### Validation of the 129-Hfe^tm.1.1Nca^ mouse model

**Fig. 2:**
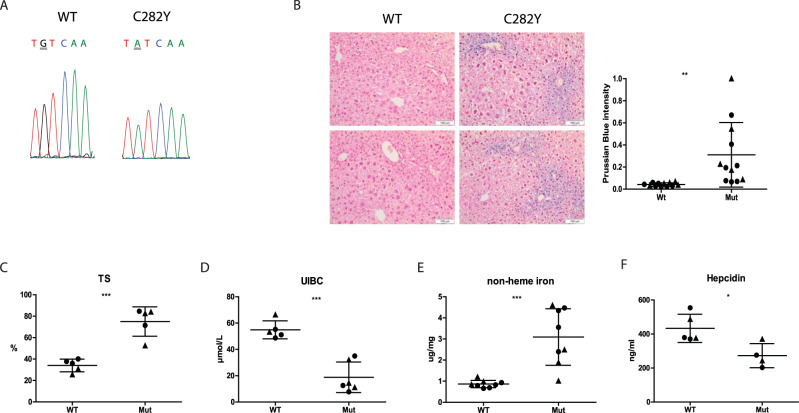
The 129-Hfe tm.1.1Nca mouse model displays increased iron accumulation. **A** Chromatogram of the *Hfe* locus obtained by Sanger sequencing of DNA of 129S6/SvEvTac or 129-Hfe tm.1.1Nca mice. The underlined nucleotides present the disease-causing mutation giving rise to C282Y. **B** Depiction of hepatic iron accumulation using Prussian Blue staining of liver sections obtained from wt or 129-Hfe tm.1.1Nca mice. Densitometric quantification was performed using ImageJ. Statistical significance was determined using a two-tailed unpaired *t* test. *p* value = 0.0043. Data are presented as mean values *+*/− SEM. Wt (129S6/SvEvTac) 0.04088 ± 0.0046, *n* *=* 12; Mut (129-Hfe tm.1.1Nca) 0.3102 ± 0.08446, *n* = 12. The bars in the scatter dot plot depict the mean with the SD. Male mice are represented in circles, female mice in triangles. **C** Transferrin saturation analysed from serum. Statistical significance was determined using a two-tailed unpaired *t* test. *p* value = 0.0003. Data are presented as mean values +/− SEM. WT (129S6/SvEvTac) 34.04 ± 2.639, *n* *=* 5; Mut (129-Hfe tm.1.1Nca) 75.00 ± 6.116, *n* *=* 5. The bars in the scatter dot plot depict the mean with the SD. Male mice are represented in circles, female mice in triangles. **D** UIBC analysed from serum. Statistical significance was determined using a two-tailed unpaired *t* test. *p* value = 0.0002. Data are presented as mean values +/− SEM. WT (129S6/SvEvTac) 54.89 ± 3.054, *n* *=* 5; Mut (129-Hfe tm.1.1Nca) 18.77 ± 4.742, *n* *=* 6. The bars in the scatter dot plot depict the mean with the SD. Male mice are represented in circles, female mice in triangles. **E** Measurement of non-heme iron levels in the liver tissue Statistical significance was determined using a two-tailed unpaired *t* test. *p* value = 0.0004. Data are presented as mean values +/− SEM. WT (129S6/SvEvTac) 0.8636 ± 0.06055, *n* *=* 8; Mut (129-Hfe tm.1.1Nca) 3.093 ± 0.4745, *n* *=* 8. The bars in the scatter dot plot depict the mean with the SD. Male mice are represented in circles, female mice in triangles and **F** hepcidin levels in serum of WT and 129-Hfetm.1.1Nca mice were compared. Both parameters show a significant difference between the two groups. Statistical significance was determined using a two-tailed unpaired *t* test. *p* value = 0.0183. Data are presented as mean values +/− SEM. WT (129S6/SvEvTac) 433.3 ± 37.13, *n* *=* 5; Mut (129-Hfe tm.1.1Nca) 272.8 ± 35.60, *n* *=* 4. The bars in the scatter dot plot depict the mean with the SD. Male mice are represented in circles, female mice in triangles. Source data are provided as a Source Data file.

The 129-Hfe^tm.1.1Nca^ mouse model was generated by introducing the human C282Y (c.845G > A) mutation into the murine *Hfe* gene^18^. The genotype was confirmed by Sanger sequencing (Fig. 2A). Compared to wild-type littermates (S129S6/SvEvTac mouse strain), the mice stored moderately more iron in the liver as measured by quantifying Prussian Blue staining (Fig. 2B), without developing liver fibrosis. Furthermore, the mice showed a higher transferrin saturation index (TS) and unbound iron binding capacity (UIBC) at 60 weeks after birth (Fig. 2C, D). In addition, elevated levels of non-heme iron in the liver as well as reduced hepcidin levels in the serum were observed in the 129-Hfetm.1.1Nca mice (Fig. 2E, F).

### An AAV8 split-vector mediates gene correction in vivo

**Fig. 3:**
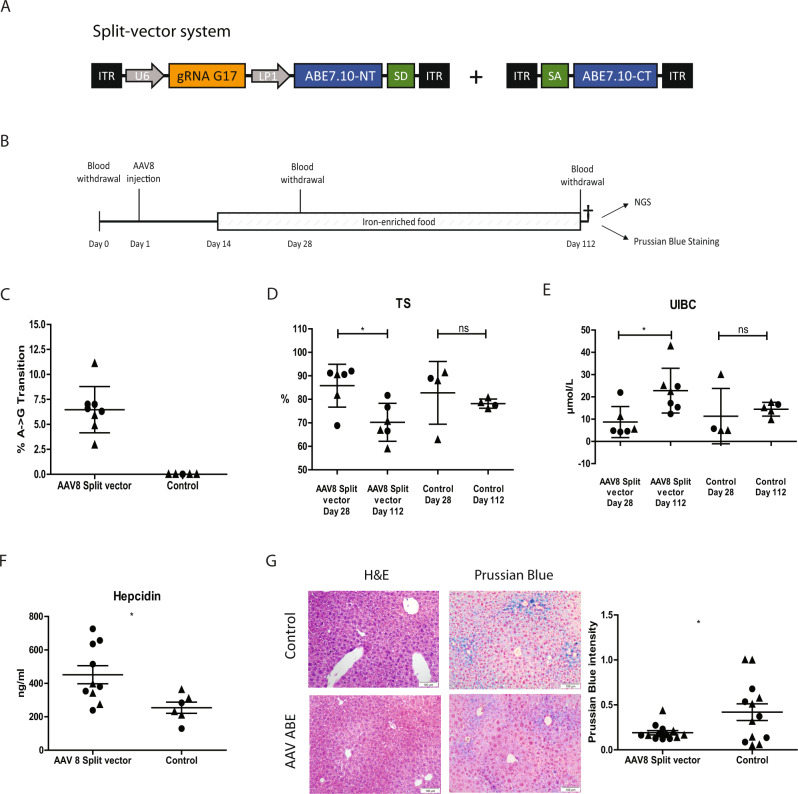
In vivo Adenine Base Editing upon low dose AAV split-vector administration. **A** Depiction of the split-vector system. AAV_SV1 contains a U6 promoter-driven gRNA gene and the LP1-driven N-terminal ABE7.10 domain. AAV_SV2 contains the C-terminal part. **B** Experimental timeline. Basal blood was withdrawn on day 0. Split-vector was injected on day 1. On day 14 animals were supplied with iron-enriched food until the endpoint. A second blood withdrawal occurred on day 28 and a third on day 112. Editing efficiency was assessed by NGS and iron accumulation was determined via Prussian Blue staining. **C** Determination of base conversion rate via NGS four months after low-dose AAV8 split-vector injection. The bars in the scatter dot plot depict the mean with the SD. Male mice are represented in circles, female mice in triangles. *Hfe* locus was PCR-amplified from liver DNA prior to NGS (*n* *=* 5 controls, *n* *=* 8 treated). **D** Transferrin saturation was measured from serum withdrawn at day 28 and 112. Statistical significance was determined using a two-tailed unpaired *t* test. *p* value = 0.0106 (AAV8 Split vector day 28 vs AAV8 Split vector day 112). Data are presented as mean values +/− SEM. TS% Day 28 85.80 ± 3.731, *n* *=* 6; TS% Day 112 70.21 ± 3.290, *n* *=* 6. The bars in the scatter dot plot depict the mean with the SD. Male mice are represented in circles, female mice in triangles. **E** UIBC was measured from serum obtained at day 28 and 112. Statistical significance was determined using a two-tailed unpaired *t* test. *p* value = 0.0147 (AAV8 Split vector day 28 vs AAV8 Split vector day 112). Data are presented as mean values +/− SEM. UIBC Day 28: 8.700 ± 2.852; *n* *=* 6; UIBC Day 112: 22.84 ± 3.798, *n* *=* 7. The bars in the scatter dot plot depict the mean with the SD. Male mice are represented in circles, female mice in triangles. **F** Hepcidin levels were measured from serum obtained at the endpoint. Statistical significance was determined using a two-tailed unpaired *t* test. *p* value = 0.0199. Data are presented as mean values +/− SEM. AAV8 Split vector: 451.7 ± 54.06, *n* *=* 10; Control: 254.2 ± 33.45, *n* *=* 6. The bars in the scatter dot plot depict the mean with the SD. Male mice are represented in circles, female mice in triangles. **G** H&E staining of liver sections and hepatic iron accumulation four months post low-dose split-vector injection. Liver sections were stained with Prussian Blue and densitometric quantification was performed Statistical significance was determined using a two-tailed unpaired *t* test. *p* value = 0.0251. Data are presented as mean values +/− SEM. AAV8 Split vector: 0.1909 ± 0.02375, *n* *=* 13; Control: 0.4191 ± 0.09250, *n* *=* 13. The bars in the scatter dot plot depict the mean with the SD. Male mice are represented in circles, female mice in triangles. Source data are provided as a Source Data file.

For the in vivo application of the adenine base editor, we took advantage of the earlier developed ABE7.10 split-vector system used to correct a dystrophin mutation^16^. The AAV8 split-vector 1 (AAV8_SV1) was equipped with a U6 promoter-driven cassette expressing our best performing gRNA (G17) and a second cassette with the hepatocyte-specific synthetic promoter (LP1), the N-terminal part of the ABE7.10 gene and the splice donor site. The AAV split-vector 2 (AAV8_SV2) contained the splice acceptor site and the remaining C-terminal portion of the ABE7.10 gene (Fig. 3A). After initial blood withdrawal from experimental animals for baseline serum parameters, both AAV8_SV1 and AAV8_SV2 were simultaneously injected at 1 × 10^11^ viral genomes (vg) per vector into the tail vein of 8- to 11-week-old mice (male/female). Control animals were injected with the same volume of saline. The timelines of the in vivo experiments are depicted in Fig. 3B. All animals were supplemented for three months with regular chow enriched with 2% carbonyl iron to achieve accelerated iron overload as described by Zhou et al.^19^. By this, we could explore whether in vivo base editing can restore iron metabolism parameters in a state of progressed hemochromatosis. 112 days post tail-vein injection of 1 × 10^11^ vg (low dose), whole-liver DNA of eight animals was analysed via NGS and revealed a base conversion frequency of 6.5% ±  2.3 (Fig. 3C). Compared to serum parameters from day 28 after AAV8 injection, the serum analysis on day 112 revealed significantly lower levels of iron transferrin saturation (TS) and higher levels of unbound iron binding capacities (UIBC). In contrast, these parameters remained unchanged in control animals (Fig. 3D, E). In addition, a significant change in hepcidin levels was detected (Fig. 3F, Supplementary Fig. 2). Prussian Blue staining of representative liver sections indicated reduced iron load in hepatocytes of treated animals, while H&E staining does not show abnormalities (Fig. 3G). Off-target activity at the top four predicted genes was analysed via NGS (Supplementary Table 3). No aberrant editing events were detected.

Taken together, the above-mentioned editing frequency led to significant changes in iron storage and related clinical parameters.

### High-dose vector application improves gene editing rate

**Fig. 4:**
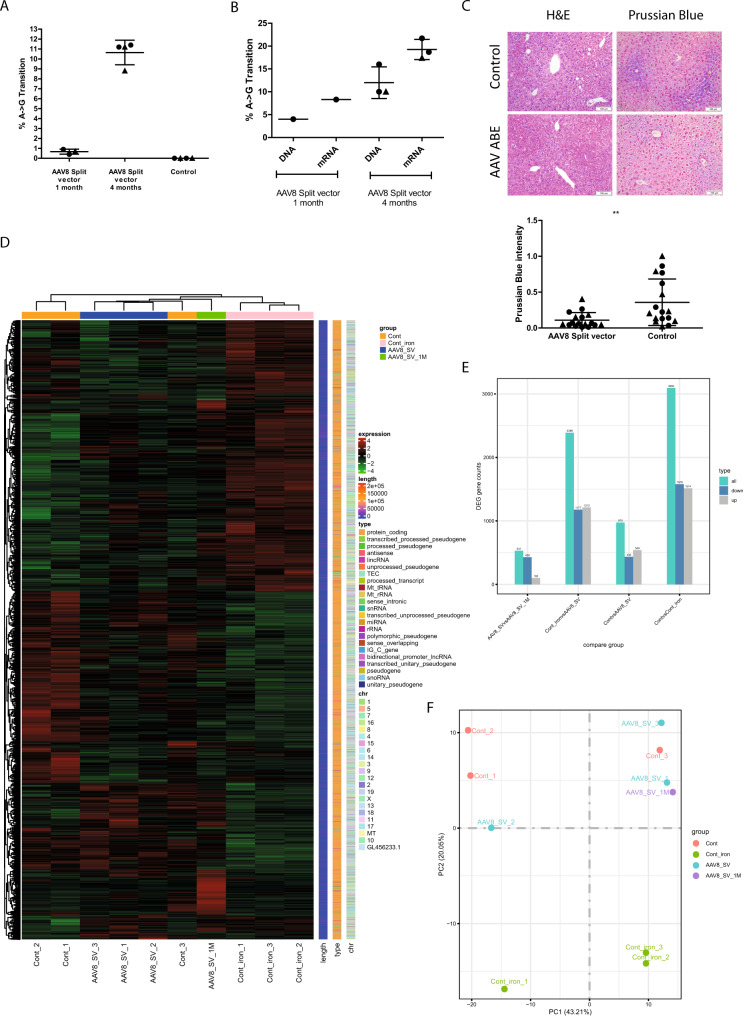
In vivo Adenine Base Editing upon high dose AAV split-vector administration. **A** Determination of base conversion rate via NGS four months after high-dose AAV8 split-vector injection in the whole liver. The bars in the scatter dot plot depict the mean with the SD. Male mice are represented in circles, female mice in triangles. *Hfe* locus was PCR-amplified from liver DNA prior to Illumina-sequencing (*n* *=* 3 controls, *n* *=* 4 treated). **B** Depiction of gene correction rate on DNA and mRNA level in isolated and highly purified hepatocytes obtained from animals one month or four months post AAV split-vector injection. The bars in the scatter dot plot depict the mean with the SD. Male mice are represented in circles, female mice in triangles. RNA sequencing of the edited Hfe locus showed 8.7% correction rate after one month and 19.7% ± 2.21 after four months (*n* = 3). Correction rates on DNA level accounted for 4% (one month) and 12% ± 3.6 (four months) (*n* *=* 1, *n* *=* 3). **C** H&E staining of liver sections and hepatic iron accumulation four months post high-dose split-vector injection. Liver sections were stained with Prussian Blue and densitometric quantification. Statistical significance was determined using a two-tailed unpaired *t* test. *p* value = 0.0049. Data are presented as mean values *+*/− SEM. AAV8 Split vector: 0.1088 ± 0.02585, *n* *=* 17; Control: 0.3586 ± 0.07858, *n* *=* 17. The bars in the scatter dot plot depict the mean with the SD. Male mice are represented in circles, female mice in triangles. **D** Overall results of FPKM cluster analysis, clustered using the log2(FPKM + 1) value. The red colour indicates genes with high expression levels, and the green colour indicates genes with low expression levels. The colour ranging from red to green indicates that log2(FPKM + 1) values wherefrom large to small. The chromosome to which each gene belongs, the gene’s length, and the biological type of the gene are also added to the heatmap. **E** Histogram showing differential gene expression based on the RNA-Seq results obtained from isolated hepatocytes of animals kept on normal food (ctrl), iron-enriched food (ctrl_iron and AAV8_SV). The number of differential genes (including upregulation and downregulation) for each comparison combination is shown in a histogram. Blue and grey represent the differential genes for upregulation and downregulation, respectively, and the numbers on the columns indicate the number of differential genes (*n* = 3 for each group, individual animal for one-month post injection). **F** Principal component analysis performed on all samples’ gene expression value (FPKM). Under ideal conditions, the samples between groups should be dispersed and the samples within groups should be gathered together. In the left half of the diagram female mice (Cont1, Cont2, AAV8_SV_2, Cont_iron_1) are located, the right half displays male mice (Cont_3, AAV8_SV_1, AAV8_SV_3, AAV8_SV_1M, Cont_iron_2, Cont_iron_3). Source data are provided as a Source Data file.

Next, we performed a series of experiments with higher titres to assess the direct effect of viral vector dose on base editing efficiency. Intriguingly, four weeks after the injection of 1 × 10^12^ vg of each AAV, the rate of editing events was very low (<1% in whole liver DNA, ~4% in hepatocyte DNA and ~8.3% hepatocyte mRNA) (Fig. 4A, B). However, four months post injection, a base conversion rate of 10.7% ± 1.2 (whole liver DNA), 12% ± 3.6 (hepatocyte DNA) and 19.3% ± 2.2 (hepatocyte RNA) (Fig. 4A, B) as well as a significant reduction of hepatic iron accumulation could be observed, while H&E staining did not show abnormalities (Fig. 4C).

To further assess the effect of a high iron diet and the efficiency of our therapeutic approach on transcriptome level, hepatocytes were isolated from animals sacrificed four months post injection and subjected to RNA-Seq analysis. Compared to the untreated normal diet controls, iron supplementation caused transcriptional changes in distinct gene clusters, which could partially be reverted after AAV8_ABE7.10 treatment as apparent from the RNA-Seq cluster analysis heatmap (Fig. 4D).

As obtained by KEGG enrichment analysis, a high iron diet has caused the enrichment of pathways such as PPAR, peroxisome and fatty acid metabolism (Supplementary Fig. 3a, Supplementary Data file 1).

More precisely, iron supplementation has caused an extensive dysregulation of 3093 (1514 upregulated, 1579 downregulated) genes (Fig. 4E). However, upon high-dose treatment with the AAV8_ABE7.10 split vector, only a set of 975 genes was dysregulated (540 upregulated, 435 downregulated) (Fig. 4E, Supplementary Fig. 3b and c) compared to untreated control animals without iron diet. This regulatory effect after gene correction was also illustrated in the principal component analysis (Fig. 4F), where AAV8_ABE7.10-treated animals, which were kept on an iron diet, showed similar transcriptomic properties as control animals without iron food. In fact, this could also be observed for a treated animal four weeks post injection. In contrast, control animals with an iron diet displayed a considerable distance from low-iron controls and treated animals (Fig. 4F), indicating a significant regulative effect.

## Discussion

In summary, we present a gene therapeutic approach for hemochromatosis based on targeted base editing of the most frequent human C282Y mutation of the *HFE* gene. Since efficacy and precision of base editing depend on the choice of the gRNA directing the ABE7.10 deaminase, we extensively tested various candidate sequences in our convenient GFP switch-on cell culture assay. In contrast to our initially established GFP switch-off system, which displayed fluctuating results, the GFP switch-on system presents a highly precise, flow cytometry-based readout tool for base editing efficiency. As a measure of the precision of this system, flow cytometry results were compared to NGS-based analysis, which led to similar results. The modular architecture of the GFP switch-on system allows the simple incorporation of a target sequence to be tested. As the readout is based on flow cytometry, it represents a cost-efficient method for gRNA screening. By creating stable cell lines holding the sequence to be targeted by ABE7.10, we could almost completely reduce background GFP expression (Supplementary Fig. 1c), which was observed in a similar transient reporter system developed by Wang and colleagues^20^.

Our in vivo experiments demonstrated a base conversion frequency of up to 10.7% ± 1.2 in whole liver DNA and significant physiological effects on iron metabolism (TS, UIBC, Hepcidin). The changes in hepcidin levels observed four months post treatment are the results of the base substitution at the *Hfe* locus and unlikely due to potential inflammation induced by AAV injection. It is known that IL-6 can activate hepcidin expression. However, as Gao et al.^21^ previously observed, AAV infection does not stimulate IL-6 secretion. To rule out the artificial elevation of hepcidin due to inflammatory processes upon AAV injection, we also measured hepcidin levels four months upon AAV8-TTR-GFP injection (Supplementary Fig. 4). No statistically significant differences in hepcidin expression were detected.

The conversion rate measured in isolated hepatocytes (DNA: 12% ± 3.6 and RNA: 19.7% ± 2.21) is slightly elevated compared to whole liver tissue. Besides vector dose, time appears to be a crucial parameter for in vivo base editing, as no substantial amounts of base conversions could be detected at four weeks post AAV injection. However, four months post injection, notable editing frequencies were achieved. Based on this observation, we hypothesised that genes involved in the DNA repair machinery in hepatocytes may act as a limiting factor for the completion of base editing. Therefore, using RNA-Seq and qRT-PCR, we carefully explored the RNA expression levels of genes mainly involved in the long patch base excision repair (BER) pathway and intriguingly we found that Ligase 1 (*Lig*1) and proliferating cell nuclear antigen (*Pcna*) were upregulated in animals four months post injection, but not one-month post injection (Supplementary Fig. 5, Supplementary Data file 2). These two factors are involved in the final steps (gap filling, strand displacement and ligation) of long patch BER as previously shown^22,23^.

Since ABE7.10 mRNA expression remained at comparable levels four months and four weeks post injection (Supplementary Fig. 6) and no increase in proliferation due to an advantage of C282Y corrected hepatocytes was detected via Ki67 staining (Supplementary Fig. 7), this finding likely explains the increasing correction rate over time by a delay of long patch BER in hepatocytes. Further experiments will be required to substantiate this hypothesis.

Experiments with longer exposure times of the base editor in the recipient hepatocytes (>one year) are currently underway to analyse whether base conversion rates will increase beyond the four-month time point and lead to complete phenotypic correction of the disease. In parallel, we will examine the transient expression of adenine base editor mRNA upon lipid nanoparticle-mediated delivery in murine hepatocytes as an alternative to a permanent AAV-mediated transgene expression.

Until then, our gene therapy approach paves the way for definitive in vivo correction of the C282Y mutation causing hemochromatosis in humans.

## Methods

The research work of this study complies with all relevant ethical regulations and the fundamental principles of good scientific practice according to the good scientific practice guidelines of Hannover Medical School.

### Plasmid construction for in vitro base-editing validation

Addgene plasmid #102919 (https://www.addgene.org/102919/) was used to express ABE7.10 for the in vitro experiments. For U6-sgRNA expression plasmids, five different gRNA oligonucleotides were synthesised (Sigma) and cloned into the pU6gRNA vector using the BsmBI restriction site (Supplementary Table 2). To generate the retroviral construct for the GFP switch-on system, *gfp* was PCR-amplified from pSERS11 SF GFP^24^ and modified by using a long primer containing Gly-Ser stretch, the stop codon TAG and the restriction site for NcoI (Sigma; P1 and P2 in Supplementary Table 1). The backbone and the PCR product were digested using NcoI and NotI (NEB) and ligated with T4 ligase (NEB).

Similarly, to generate the retroviral construct for the HFE-GFP switch-on system, *gfp* was PCR-amplified from pSERS11 SF GFP using a long primer containing 20 bp of the *HFE* sequence, carrying the stop codon TAG, a Gly-Ser stretch and the restriction site for NcoI (Sigma; P3 and P2 in Supplementary Table 1). The backbone and the PCR product were digested using NcoI and NotI (NEB) and ligated with T4 ligase (NEB). Amplification of plasmids was performed in One Shot™ Stbl3™ Chemically Competent *E. coli* (C737303, Invitrogen).

### Cell line generation

The GFP switch-on system and the HFE-GFP switch-on system plasmids were packaged into retrovirus particles by co-transfecting HEK-293T cells with pMLV gag-pol and pVSV-G as previously described in Schambach et al.^24^ using Lipofectamine 3000 (Invitrogen). Three days post transfection, the supernatant was harvested and centrifuged for 45 min at 1500 × *g*, at 4°. The pellet was diluted in 100 µl PBS (Gibco) and filtered using 0.45 µm filters (Sarstedt).

HEK-293T cells were directly transduced using the retroviruses to generate HEK-293T-GFP_TAG and HEK-293T-HFE_TAG_GFP stable cell lines. The resulting polyclonal cell populations underwent limiting dilution to isolate single monoclonal cell populations.

### Cell culture and transfection

HEK-293T (ATCC), HEK-293T- GFP_TAG and HEK-293T- HFE_TAG_GFP cells were maintained in antibiotic-free DMEM (Thermo Fisher Scientific) supplemented with 10% (v/v) fetal bovine serum (Thermo Fisher) and 1% Pen-Strep (Gibco) at 37 °C with 5% CO_2_.

HEK-293T_GFP_TAG and HEK-293T_HFE_TAG_GFP cells were seeded in 12-well plates at a concentration of 2 × 10^5^ cells/ml. 0.6 μg of gRNA-expressing plasmid and 1.2 μg of pCMV-ABE 7.10 constructs were co-transfected with 2 μl of Lipofectamine 3000. The cells were expanded and transferred into six-well plates.

Cells were analysed for GFP expression using a fluorescence microscope (Nikon Eclipse TS100) 48, 72 and 96 h post transfection. Quantification of GFP^+^ cells was performed 96 h post transfection by flow cytometry.

### Flow cytometry

Four days post-transfection, HEK-293T_GFP_TAG and HEK-293T_HFE_TAG__GFP cells transfected with ABE7.10 plasmids were collected from six-well plates and analysed on BD FACSCalibur (BD Biosciences) to determine GFP-positive cells. A number of at least 100,000 cell events were collected, and data analysis was performed using the FlowJo software (Tree Star).

### Virus production for in vivo delivery of ABE agents

Addgene plasmid #112734 (https://www.addgene.org/112734/) was modified using NEBuilder HiFi DNA Assembly (NEB #E2621). The vector was digested with NdeI (NEB) and EcoRI (NEB) and the inserts gRNA G17 (adapted for the mouse sequence, in Supplementary Table 2; primers P4 and P5 in Supplementary Table 1) and LP1 promoter (pSSV9_LP1_iCaspase9 as described in Krooss et al.^4^; primers P6 and P7 in Supplementary Table 1) were amplified via PCR. pAAV-ABE-NT-gRNA (pAAV_SV1) and Addgene plasmid #112876 (https://www.addgene.org/112876/) (pAAV_SV2) were prepared using EndoFree Plasmid Maxi Kit (Qiagen) and the integrity of ITRs was checked via SmaI (NEB) digestion.

For the production of AAV vectors, HEK-293 cells were seeded at 80% confluency and co-transfected with the vector plasmid (pAAV_SV1 or pAAV_SV2) and pDP8.ape (PlasmidFactory), which combines AAV8 packaging and Adenohelper genes, at a 1:1 molar ratio. Forty-eight hours post-transfection, cells were harvested, pelleted by low-speed centrifugation and lysed. Vectors were purified from cell lysates by CsCl density gradient centrifugation as described^25^. Genomic titer was determined by quantitative qPCR following isolation of vector genomes using specific primers P8 and P9 for AAV_SV1 and P10 and P11 for AAV_SV2, listed in Supplementary Table 1. SIRION Biotech performed the production for the high-dose experiment.

### Mouse experiments

Animal handling and experiments were performed according to the guidelines of the Hannover Medical School, Hannover, Germany, and with the permission of the local authorities (TVA 18/2876). 8- to 11-weeks-old (https://www.jax.org/strain/005063) (The Jackson Laboratory) male and female mice were used for all studies. Genotyping was performed using standard PCR methods with the primers oIMR3645 and oIMR3646, listed in Supplementary Table 1. Mice were injected (tail vein) with AAV8_SV1 and AAV8_SV2 at the indicated dose. The animals were euthanised one month and four months post treatment. Blood and individual tissues were collected for further analysis. The total number of experimental animals in this study amounts to 58.

### Histology

Livers were fixed with Roti-Histofix 4%, embedded in paraffin, sectioned at 3 μm and stained with Iron Stain Kit (ab150674). Liver sections were deparaffinised, rehydrated and stained according to the manufacturer’s protocol. Prussian Blue Stain was performed using Iron Stain Kit (ab150674) according to the manufacturer’s protocol. To quantify the size of Prussian Blue-positive areas, the stitched images were imported into ImageJ (Version 1.50e) and their threshold was adjusted accordingly. Afterwards, the Prussian Blue areas were counted using the plug-in “analyse particles”. Liver sections were also used to perform H&E staining (Carl Roth, Art. No. 9194.1). Deparaffinisation, rehydration and staining were carried out according to the manufacturer’s protocol.

### Next-generation sequencing

HEK-293T_GFP_TAG and HEK-293T_HFE_TAG_GFP cells were collected five days post transfection and DNA was extracted using DNeasy Blood & Tissue kit (Qiagen). Whole-liver DNA of treated and control animals was extracted similarly. PCR on the target locus was performed using Q5 High-Fidelity polymerase (NEB) and primers listed in Supplementary Table 1 (P12 and P13 for HEK-293T_GFP_TAG and HEK-293T_HFE_TAG_GFP; oIMR3645 and oIMR3646 for the animal samples). The PCR products were purified via QIAquick gel extraction kit (Qiagen) and employed for next-generation sequencing (NGS) analysis. To avoid bias by over-amplification, library preparation was performed using the NEBNext Ultra II DNA Library Prep Kit with a minimum of three PCR cycles (New England Biolabs). Libraries were sequenced on a MiSeq Sequencer (Illumina) using MiSeq Reagent Kit v3 (Illumina) to generate 2 × 300 base paired-end reads. Reads were trimmed using BBDuk and paired-end Illumina reads were mapped to the reference sequence using Bowtie2^26^ local alignment. Variant calling and quantification were performed via Geneious Prime 2021 1.1.

### RNA-Seq of isolated hepatocytes

Hepatocytes were isolated from 129-Hfe tm.1.1Nca mice (untreated no-iron controls, untreated iron controls and treated) by a modified 2-step Liberase perfusion^27,28^. Briefly, mice were anaesthetised using Ketamin (Albrecht, Germany) and Rompun (Bayer, Germany). The body cavity was opened and a catheter was placed into the vena cava and connected to a flow pump, which pumped media pre-warmed at 37 °C from a water bath into the catheter, and the portal vein was cut. The liver was first perfused with Earle’s Balanced Salt Solution (EBSS) (GIBCO, Germany) solution containing 0.5 mM Ethylene Glycol Tetra acetic acid (EGTA) (Sigma-Aldrich Germany) and 10 mM HEPES buffer (Sigma Germany). Subsequently, EBSS supplemented with 10 mM HEPES buffer and Liberase (Roche, Germany) 100 µg/L was applied for enzymatic tissue digestion at 37 °C. After digestion of 10–12 minutes, the liver was carefully disconnected and tissue was manually disrupted with sterile scissors and scalpel in Dulbecco’s Modified Eagle’s Medium (DMEM) (GIBCO Germany) containing 10% FCS. The suspended hepatocytes were passed through a 100 µm nylon filter into 50 ml Falcon tubes. The cell suspensions were centrifuged twice at 300 × *g* for 5 min at 4 °C and the cell pellet was resuspended in 15 ml ice-cold DMEM medium containing 10% fatal calf serum (FCS) (PAN biotech Germany).

After isolation, hepatocytes were purified using Percoll (Cytiva). Three 10 ml tubes for each mouse were filled with 5 ml of 50% Percoll solution, then 2 ml of 24% Percoll solution was added on top of it and finally 5 ml of cell suspension was carefully layered up. The gradient was centrifuged at 1000 × *g*, for 30 min at 4°. The pellet was then resuspended in 10 ml of DMEM and centrifuged at 50 × *g*, for 5 min at 4°. Finally, the cells were resuspended in DMEM and the cell viability was tested by trypan blue (Fluka Germany).

Total RNA was purified using the Macherey-Nagel RNA purification kit according to the manufacturer’s protocol. RNA-Seq analysis including sample quality control, library construction, sequencing, read mapping, differential expression analysis was performed by Novogene. Mapping was performed using hisat2 2.0.5, quantification using featureCounts 1.5.0-p3, differential analysis with DESeq2 1.20.0 for samples with biological replicate and edgeR 3.22.5 for samples without biological replicates, enrichment analysis using clusterProfiler 3.8.1. Differentially expressed genes between two samples were analysed using Geneious Prime® 2021.1.1. Reads were mapped to the reference shown in Supplementary Data file 2. The number of transcripts was compared and normalised by the Median of Gene Expression Ratios as described by Dillies et al.^29^.

### Serological analysis

Serum iron and unbound iron binding capacity (UIBC) were measured by Iron Direct Method kit (Ferene) and Iron UIBC kit (Biolabo), respectively. Transferrin saturation is the ratio (%) of plasma iron concentration to total iron binding capacity. In additional, serum hepcidin concentration was evaluated by an ELISA assay (HMC-001, Intrinsic Sciences), following the manufacturer’s instructions.

### Statistical analysis

Statistical analyses were performed using GraphPad Prism (v.5.01). All described experiments were conducted using at least biological triplicates. Standard error of mean (SEM) was calculated and different categories were compared using two-tailed unpaired *t* test. Statistical significance was considered with a *p*-value of <0.05.

### Reporting summary

Further information on research design is available in the Nature Research Reporting Summary linked to this article.

## Supplementary information

Supplementary Information

Reporting Summary

Description of Additional Supplementary Files

Supplementary Dataset 1

Supplementary Data File 2

## Data Availability

The datasets generated during and/or analysed during the current study are available from the corresponding author on reasonable request. Source data are provided with this paper. The RNA-Seq data and NGS data reported in this study have been deposited in the Genome Sequence Archive (Genomics, Proteomics & Bioinformatics 2021) in National Genomics Data Center (Nucleic Acids Res 2021), China National Center for Bioinformation/Beijing Institute of Genomics, Chinese Academy of Sciences (GSA: CRA011170 - RNA-Seq data - and CRA011176 - NGS data) that are publicly accessible at https://ngdc.cncb.ac.cn/gsa^30,31^. The supplementary figures and tables generated in this study are provided in the Supplementary Information, Supplementary Data files and in the Source Data file. Source data are provided with this paper.

## Source data

Source Data
