# Supplementary material for: In vivo adenine base editing reverts C282Y and improves iron metabolism in hemochromatosis mice
Source: Nat Commun. 2022 Sep 5;13:5215. doi: 10.1038/s41467-022-32906-9 (PMC9445023; doi:10.1038/s41467-022-32906-9)
Supplement: Supplementary file 1 — Supplementary Information [file 41467_2022_32906_MOESM1_ESM.pdf]

Supplementary Information

Supplementary Figure 1

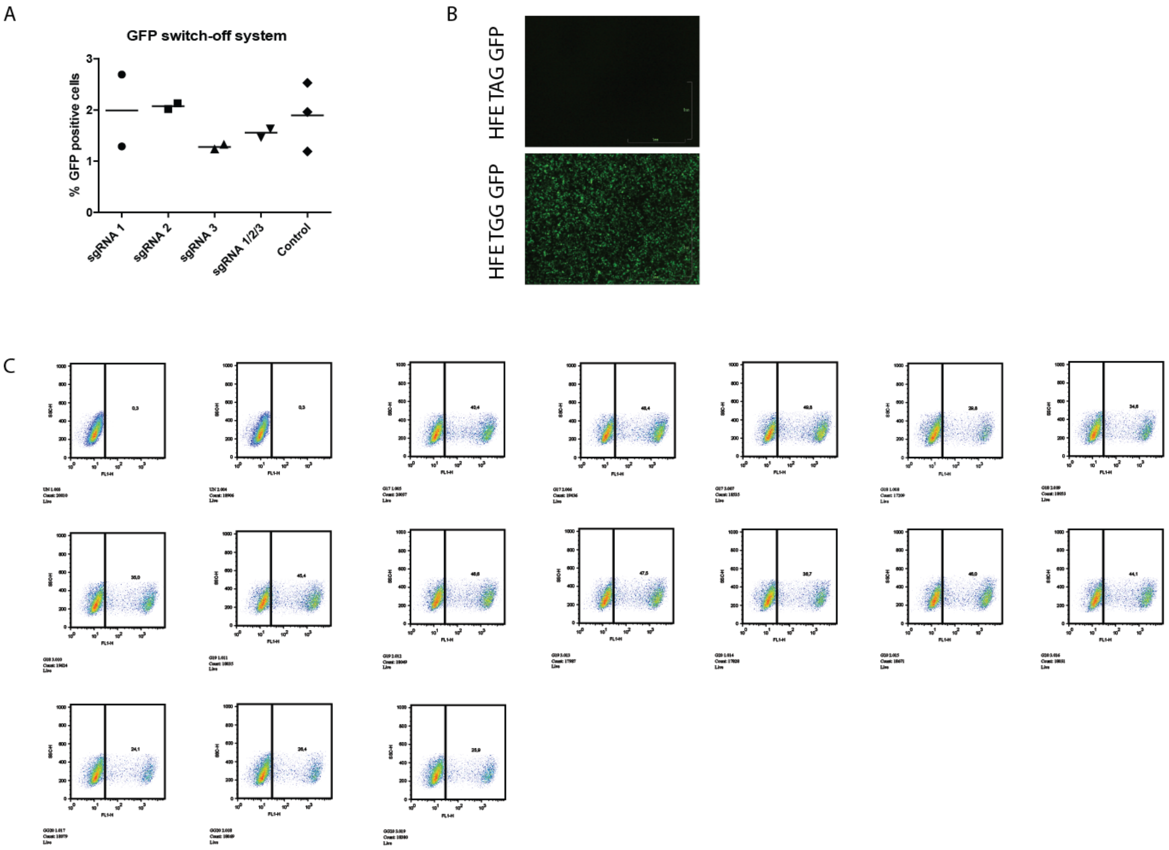

Supplemental Figure S1| **Insight into the cell culture-based base editing reporter systems**

A) Flow cytometric analysis of retrovirally transduced HEK-293T cells harboring the GFP switch-off system. Cells were transfected with various gRNAs and ABE7.10 plasmid (n=2). The bars in the scatter dot plot depict the mean. **B)** Fluorescence microscopy (GFP x 10) of HEK-293T cells transfected with the HFE-GFP switch-on system. The upper picture displays cells harboring the *HFE* sequence with a stop codon and GS-linker in front of the *gfp*, avoiding GFP expression. The lower picture shows unrestricted GFP function upon modification of the TAG stop codon to TGG. This experiment was performed in two biological replicates and showed nearly identical results. **C)** Raw data of flow cytometric analysis of HEK-293T cells retrovirally transduced with the GFP switch-on system. Untreated cells present no GFP expression, whereas gRNA + ABE7.10 transfected cells are GFP-positive. Source data are provided as a Source Data file.

Supplementary Figure 2

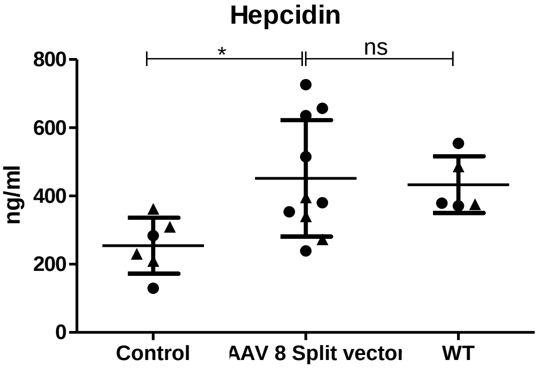

Supplemental Figure S2| **Evaluation of hepcidin levels in untreated, treated and WT mice**

Hepcidin levels were measured from serum obtained at the endpoint. Statistical significance was determined using a two-tailed unpaired t test. p value= 0.0199 (Control vs AAV8 Split vector); p value= 0.8258 (AAV8 Split vector vs WT). Data are presented as mean values +/- SEM. Control: 254.2 ± 33.45, n=6; AAV8 Split vector: 451.7 ± 54.06, n=10; WT 433.3 ± 37.13, n=5. The bars in the scatter dot plot depict the mean with the SD. Male mice are represented in circles, female mice in triangles. Source data are provided as a Source Data file.

Supplementary Figure 3

A

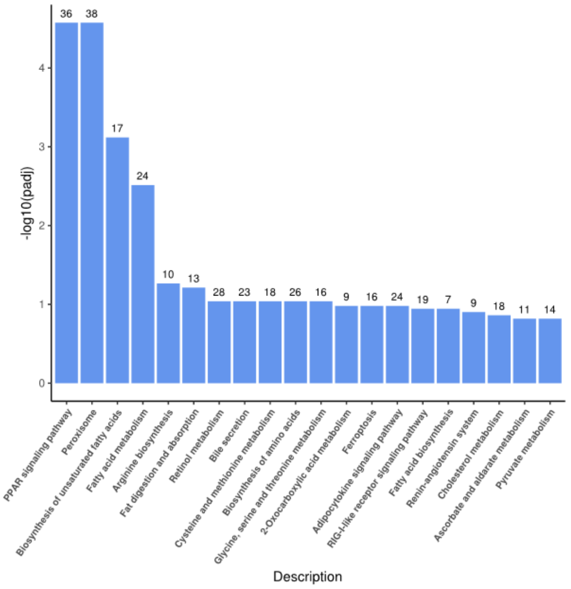

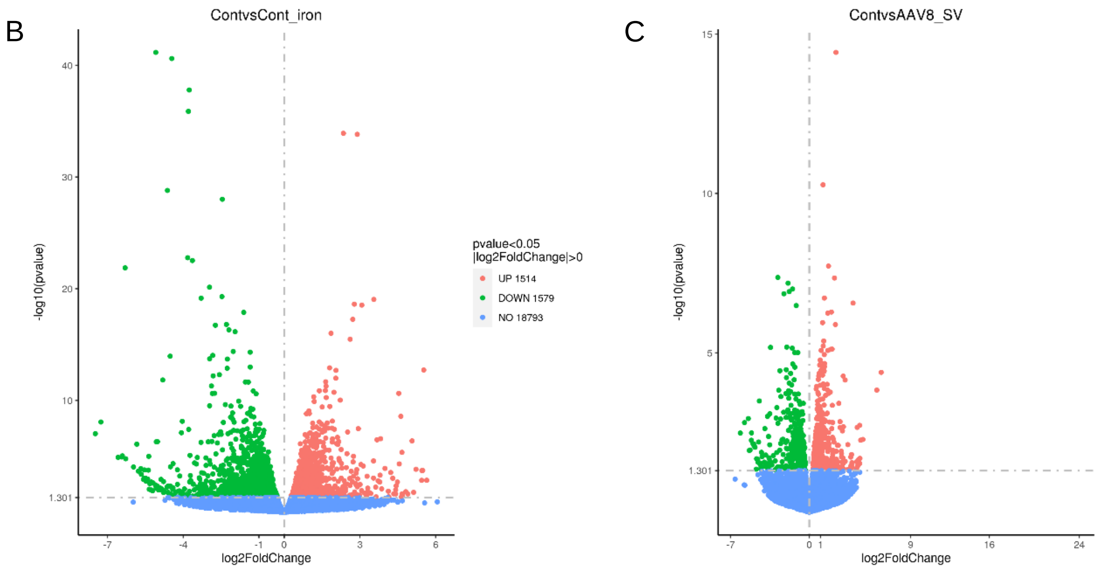

Supplemental Figure S3 | **Gene expression analysis of differential expression genes**

A) KEGG enrichment analysis in hepatocytes obtained from 129-Hfe tm.1.1Nca mice kept on normal diet vs high iron diet. In the KEGG enrichment results, the most significant 20 KEGG pathways were selected for display. In this figure, the abscissa is the KEGG pathway, and the ordinate is the significance level of the pathway enrichment. Higher values correspond to higher significance. The Volcano Map in **B)** compared the DEGs between control animals on normal and on high-iron diet, while the Volcano Map in **C)** showed the comparison between controls and AAV8-treated animals on high-iron diet. The representations are as follows: the abscissa is logFC; the ordinate -log10 of a p-value. Every dot represents a gene. In red are the dots with the p-values < 0.05 and the logFC > 0, in green the dots with the p-values < 0.05 and the logFC < 0. Blue dots indicate the remaining genes present in the array that were not significantly changed. The genes that are upregulated in the array are on the right panel, and downregulated ones are on the left panel of the plot. Data was obtained from 3 animals per group (n=3).

Supplemental Figure S4

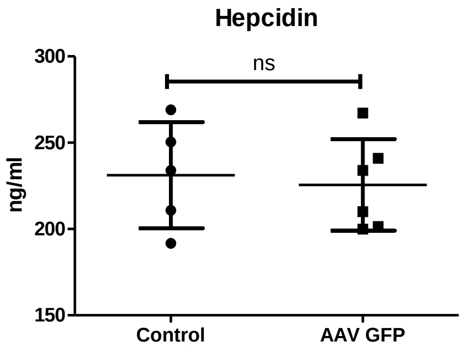

Supplemental Figure S4 | **Evaluation of alterations in hepcidin levels upon AAV injection**

C57/BL6 mice were injected or not with 1×10^{12} viral genomes of AAV8-TTR-GFP vector. Four months post injection, serum was collected and hepcidin levels were determined. Statistical significance was determined using a two-tailed unpaired t test. p value= 0.7531, not significant. Data are presented as mean values +/- SEM. Control: 231.2 ± 30.75, n=5; AAV GFP: 225.6 ± 26.53, n=6. The bars in the scatter dot plot depict the mean with the SD. Source data are provided as a Source Data file.

Supplementary Figure 5

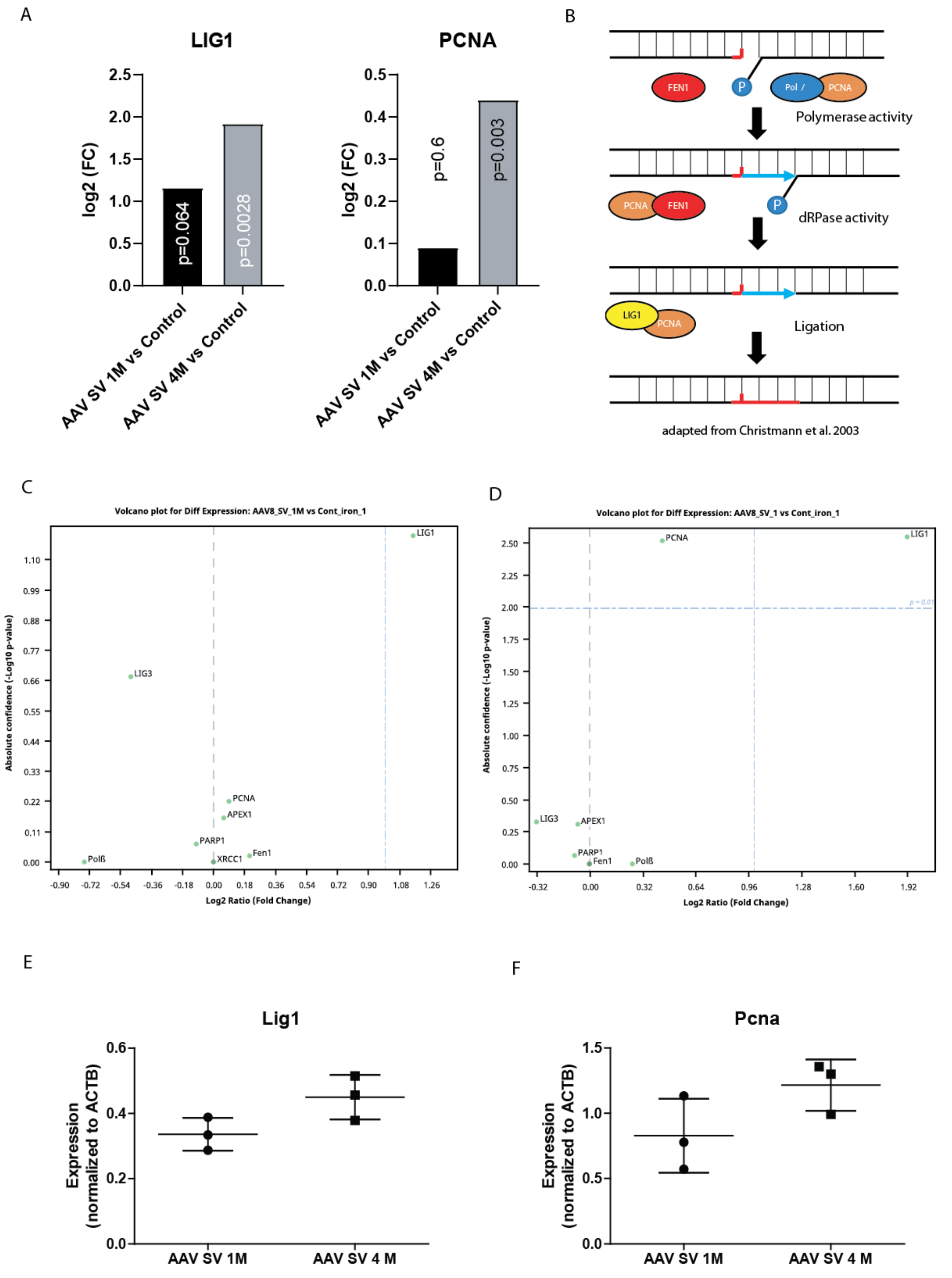

Supplemental Figure S5| **Gene expression analysis of Lig1 and Pcna in one-month and four-month treated mice**

A) Depiction of Ligase 1 and Pcna mRNA expression in log2 fold change. Calculation was performed using the basic differential gene expression plugin from Geneious Prime® 2021.1.1. The differential expression p-value is provided in each column. Expression differences to control animals are depicted one month (left column) or four months post injection. **B)** Schematic of the final steps of long patch base

excision repair (BER), adapted from Christmann et al. 2003. **C)** Volcano Plot depicting Log2 Ratio (FC) and Absolute confidence (-Log10 p-value) of genes involved in long patch BER. Animals one month post AAV8_ABE7.10 injection compared to control animals. **D)** Volcano Plot depicting Log2 Ratio (FC) and Absolute confidence (-Log10 p-value) of genes involved in long patch BER. Animals four months post AAV8_ABE7.10 injection compared to control animals. **E)** qRT-PCR analysis of Lig1 and **F)** of Pcna expression performed on liver-RNA obtained from animals one or four months post AAV8_ABE7.10 injection using primers P22+P23 (Lig1) and P24+P25 (Pcna), normalised to ACTB (n=3 biological replicates). The bars in the scatter dot plot depict the mean with the SD. Source data are provided as a Source Data file.

Supplementary Figure 6

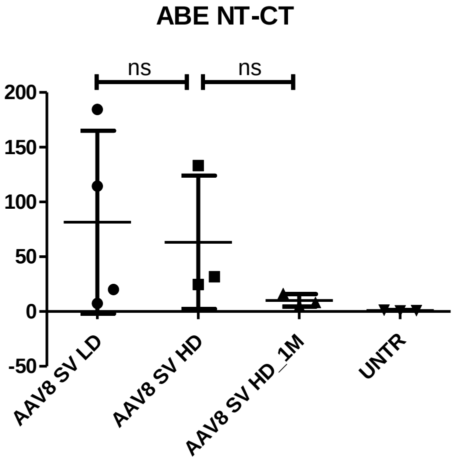

Supplemental Figure S6 | **ABE7.10 expression in liver samples of treated and untreated mice**

qRT-PCR analysis to assess ABE7.10 expression in liver-RNA obtained from animals injected with AAV8 low dose, AAV8 high dose, AAV8 high dose one month or untreated. No significant differences in ABE7.10 expression were detected using a two-tailed unpaired t test among the injected animals (AAV8 SV LD n=4; AAV8 SV HD n=3; AAV8 SV HD_1M n=3; UNTR n=3). The bars in the scatter dot plot depict the mean with the SD. Source data are provided as a Source Data file.

Supplementary Figure 7

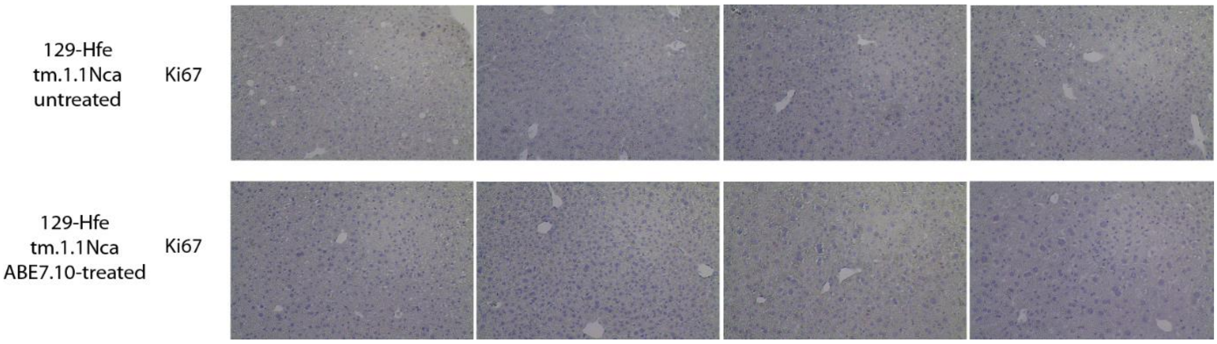

Supplemental Figure S7 | **Ki67 staining in liver samples of untreated and treated mice**

Ki67 staining in liver sections obtained from untreated animals (upper panel) and AAV8_ABE7.10 injected animals (lower panel). No enrichment of Ki67 could be detected. Liver sections were obtained from four different animals of each condition (n=4). All stainings showed comparable results. Representative stainings are depicted. Scale bar: 100 µm.

Supplementary Figure 8

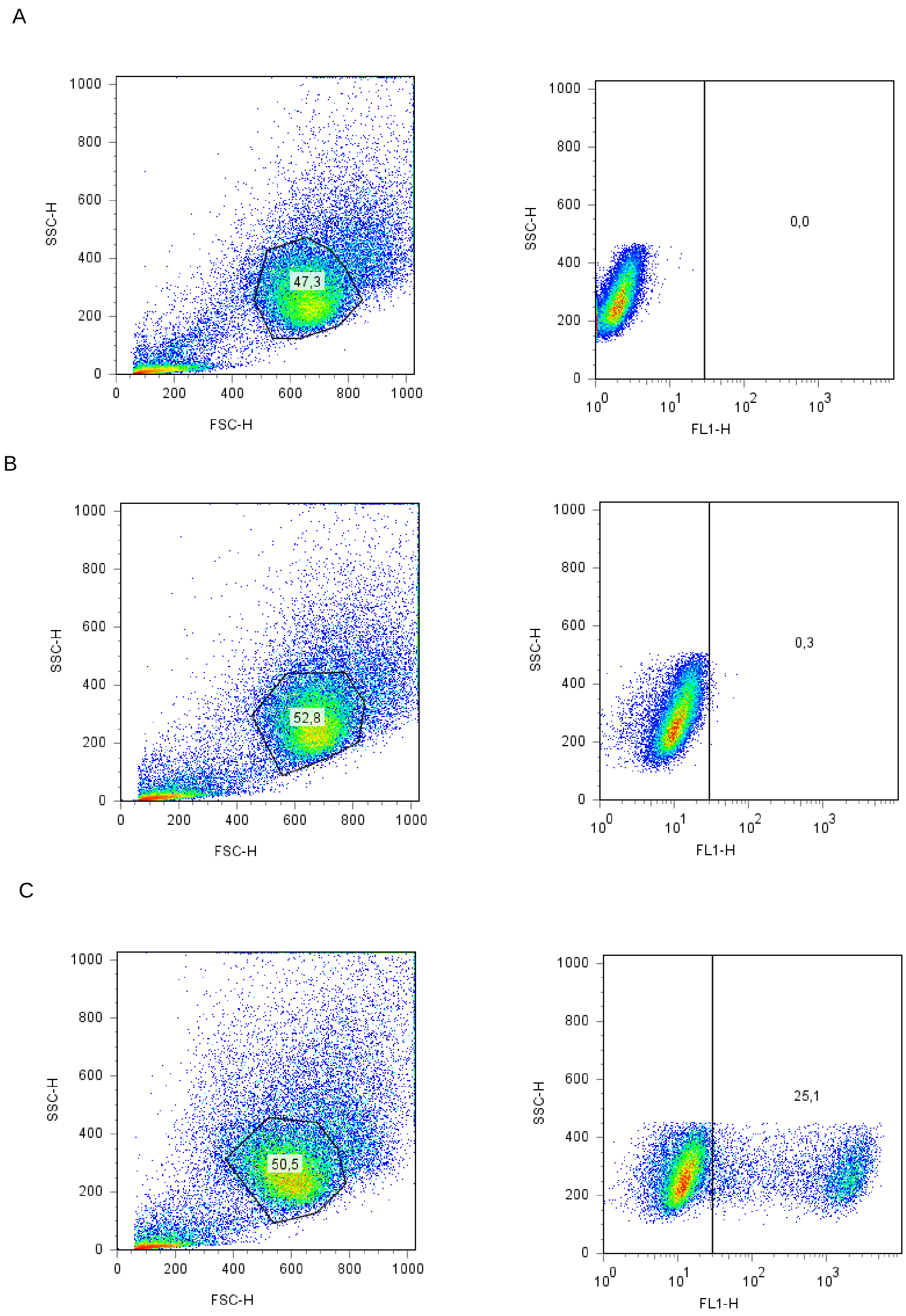

Supplemental Figure S8 | **Flow cytometry gating strategy**

Alive cells were identified based on forward (FSC-H) and side scatter (SSC-H) and the selected cells were then gated based on the side scatter (SSC-H) and the GFP expression (fluorescence intensity

FL1-H). In **A)** it is shown the gating for untreated HEK-293T cells, in **B)** for the untreated HFE-GFP switch-on cell line and in **C)** for ABE7.10 plus gRNA treated HFE-GFP switch-on cells.

Supplementary Table 1

**Primers**	**Sequence**
oIMR3645	ATCAAGGCTGGCTGACATTG
oIMR3646	ATGGCCTGAGATTGCAAGG
P1	CACCATGGGATCAGGATCAGGCTCGTAGTCGGGCTCAGGTAGGGTGAGCAAGGG CGAGGAGCTGTTCAC
P2	CGACGCGGCCGCTTTACTTG
P3	ATCCACCGGTCGCCACCATGGGCACGTaGCAGGTGGAGCACCCAGGCGGAGGCT CAGGAGGCGGATCAGGAGGCGTGAGCAAGGGCGAGGAGCTGTTCAC
P4	attatgttttaaaatggac
P5	cggacggccgccaccgcggtggctAAAAAAGCACCGACTCGGTG
P6	agccaccgcggtggcggccgtccgCCCTAAAATGGGCAAACATT
P7	CTCATCCAATACTCGTGGGAGAATTCCACTTCACTCGCTTCTACCTTTCTCTTCTTT TTTGGCATggtggcgctagcctagaatt
P8	GGGCCTATTTCCCATGATTCCT
P9	AAAACTGCAAACTACCCAAGAAA
P10	GAGATCGTGTGGGACAAGGG
P11	GATCAGCTTGTCGCTGTTGC
P12	TTCTGTTCGCGCGCTTCTGCT
P13	CCTCGCCCTCGCCGGACA
P14	CTGACTGGCAAACACAGCAGA
P15	TCATTGAGGTCATGGGGGTAAT
P16	CTGCTGTGTGAGAGAGGAGC
P17	AGGCAGGTATTTGGTCAGCA
P18	GCCAGGCATTTGTGAGAAGC
P19	CTCGACACAACCAAAGGTGC
P20	TCTCTGGGGTTGTTTGGATATGG
P21	CACAGTTCTGAATGTCCATGACTA
P22	LIG1_fw_TCCGTAAAGACAAGCAGCC
P23	LIG1_rev_ACACACACCAATCAACACC
P24	PCNA_fw_TTCACAAAAGCCACTCCAC
P25	PCNA_rev_GCAATGCCTAAGATGCTTCC

Supplemental Table 1 | **Primer sequences**

Sequences of the primers used to amplify the targeted loci for Sanger sequencing or NGS.

Supplementary Table 2

sgRNA	Sequence
sgRNA GFP TAG 1	GTCGTAGTCGGGCTCAGGTA
sgRNA GFP TAG 2	GCTCGTAGTCGGGCTCAGGT
sgRNA1 GFP switch-off	GAACCATGGTGAGCAAGGGC
sgRNA2 GFP switch-off	GCAACCATGGTGAGCAAGGGC
sgRNA3 GFP switch-off	GACCATGGTGAGCAAGGGC
G19 human	GCGTAGCAGGTGGAGCACCC
G17 human	GTAGCAGGTGGAGCACCC
G18 human	GGTAGCAGGTGGAGCACCC
G20 human	GACGTAGCAGGTGGAGCACCC
GG20 human	GGACGTAGCAGGTGGAGCACCC
G17 mouse	GTATCAAGTGGAGCACCC

Supplemental Table 2 | **gRNA sequences**

List of gRNA sequences used in the study

Supplementary Table 3

Reference Sequence	Replicate	Genomic Locus analycsed	Primers	Sample	Results	Reads
Chr1	1	chr1:76,996,658-76,996,958	P14+P15	S1	No SNPs	199,400
Chr1	2			S6	No SNPs	194,283
Chr1	3			S11	No SNPs	190,000
Chr8_1	1	chr8:25,574,115-25,574,427	P16+P17	S2	No SNPs	173,814
Chr8_1	2			S7	No SNPs	167,822
Chr8_1	3			S12	No SNPs	183,626
Chr8_2	1	chr8:115,338,273-115,338,562	P18+P19	S3	No SNPs	136
Chr8_2	2			S8	No SNPs	10
Chr8_2	3			S13	No SNPs	42
Chr10	1	chr10:116,590,393-116,590,642	P20+P21	S4	No SNPs	231,704
Chr10	2			S9	Ref-Chromosome 10 A **243** G -> A 212,968 SNP (transition) G 0.2 % A	214,245<br>**PCR AMPLIFICATION ARTIFACT**
Chr10	3			S14	No SNPs	192,000

Supplemental Table 3 | **Off-Target Site Analysis**

Off-target analysis was performed using Benchling and CasOFFinder. The first four hits from each program were consistent and the resulting list is a combination of both, each starting with a minimum of two mismatches. Based on these results, PCR primers (P14-P21) were designed to amplify the according regions. PCR products were then subjected to Illumina sequencing, reads were mapped to the corresponding reference sequences and nucleotide change as well as variant frequency was analysed using Geneious Prime® 2021.1.1.
