# Supplementary material for: In vivo adenine base editing reverts C282Y and improves iron metabolism in hemochromatosis mice
Source: Nat Commun. 2022 Sep 5;13:5215. doi: 10.1038/s41467-022-32906-9 (PMC9445023; doi:10.1038/s41467-022-32906-9)
Supplement: Supplementary file 3 — Description of Additional Supplementary Files [file 41467_2022_32906_MOESM3_ESM.pdf]

Title: Supplementary Data 1:

Description: KEGG enrichment analysis results.

KEGG enrichment analysis performed in hepatocytes obtained from 129-Hfe tm.1.1Nca kept on normal diet vs high iron diet. RNAseq analysis was performed by Novogene.

Title: Supplementary Data 2:

Description: Reference Sequence used for differential gene expression analysis of BER-involved genes.

Differentially expressed genes between two samples were analysed using Geneious Prime® 2021.1.1. Reads were mapped to the reference shown above, each gene including its NCBI reference sequence and the predicted mRNA sequence is shown in a separate tab. The number of transcripts was compared and normalised by the Median of Gene Expression Ratios as described by Dillies et al. 2013.
